# A Testimony of the Surgent SARS-CoV-2 in the Immunological Panorama of the Human Host

**DOI:** 10.3389/fcimb.2020.575404

**Published:** 2020-10-16

**Authors:** Rinki Minakshi, Arif Tasleem Jan, Safikur Rahman, Jihoe Kim

**Affiliations:** ^1^Department of Microbiology, Swami Shraddhanand College, University of Delhi, New Delhi, India; ^2^School of Biosciences and Biotechnology, Baba Ghulam Shah Badshah University, Rajouri, India; ^3^Munshi Singh College, BR Ambedkar Bihar University, Muzaffarpur, India; ^4^Department of Medical Biotechnology, Research Institute of Cell Culture, Yeungnam University, Gyeongsan-si, South Korea

**Keywords:** SARS-CoV-2, receptor binding domain (RBD), angiotensin-converting enzyme 2 (ACE-2), transmembrane protease serine 2 (TMPRSS2), acute respiratory distress syndrome (ARDS), cytokine storm, inflammatory cytokines, innate immunity

## Abstract

The resurgence of SARS in the late December of 2019 due to a novel coronavirus, SARS-CoV-2, has shadowed the world with a pandemic. The physiopathology of this virus is very much in semblance with the previously known SARS-CoV and MERS-CoV. However, the unprecedented transmissibility of SARS-CoV-2 has been puzzling the scientific efforts. Though the virus harbors much of the genetic and architectural features of SARS-CoV, a few differences acquired during its evolutionary selective pressure is helping the SARS-CoV-2 to establish prodigious infection. Making entry into host the cell through already established ACE-2 receptor concerted with the action of TMPRSS2, is considered important for the virus. During the infection cycle of SARS-CoV-2, the innate immunity witnesses maximum dysregulations in its molecular network causing fatalities in aged, comorbid cases. The overt immunopathology manifested due to robust cytokine storm shows ARDS in severe cases of SARS-CoV-2. A delayed IFN activation gives appropriate time to the replicating virus to evade the host antiviral response and cause disruption of the adaptive response as well. We have compiled various aspects of SARS-CoV-2 in relation to its unique structural features and ability to modulate innate as well adaptive response in host, aiming at understanding the dynamism of infection.

**Graphical Abstract:**
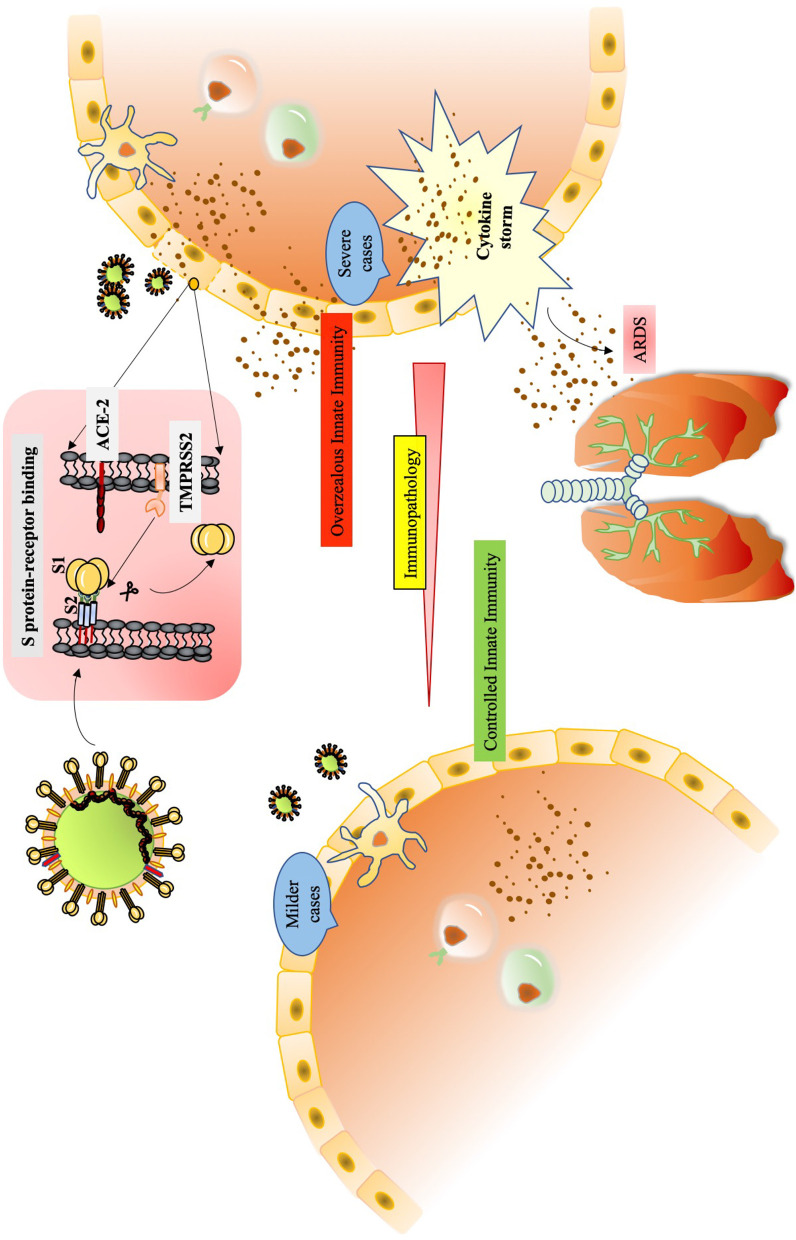
Pathophysiology of SARS-CoV-2.

## Introduction

The ever-increasing number of perilous alterations of wildlife through various human interventions have presented the global face of humanity with growing experiences of several infectious outbreaks. A protracted meeting between humans and bats has presented a timeline of pandemics caused by the coronaviruses (Cui et al., 2007; Fan et al., 2019; Sifuentes-Rodríguez and Palacios-Reyes, 2020). History now tells of the 2002–2003 Severe Acute Respiratory Syndrome (SARS) and 2011 Middle East Respiratory Syndrome (MERS), both being zoonotically originated infections in humans that were caused by new coronaviruses, SARS-CoV and MERS-CoV, respectively (Minakshi et al., 2014; de Wit et al., 2016; Song et al., 2019; Prompetchara et al., 2020; Rabaan et al., 2020). A new addendum to this is the late 2019 outbreak due to another novel coronavirus, SARS-CoV-2, that caused illness related to the respiratory system (Guo et al., 2020). The emergence of SARS-CoV-2 is putting tremendous pressure on the international community wherein we are witnessing the unprecedented lockdown imposed in various countries across the globe.

## Knowing SARS-CoV-2

The late December of 2019 witnessed cases of respiratory infection symptoms like cough, fever and dyspnea that led to pneumonia among clusters of patients in Wuhan (China). The condition was intriguing as the causative agent was unknown thus driving the medical community to search for the reason (Zhu et al., 2020). This investigation led to the isolation of a “novel” coronavirus, which was identified through next-generation sequencing and given a provisional name, the 2019 novel coronavirus (2019-nCoV) (Lu et al., 2020).

Out of the four genera of coronaviruses, α, β, γ, and δ, the human coronaviruses (HCoVs) fall in the genus α (HCoV-229E and NL63) and the genus β (MERS-CoV, SARS-CoV, HCoV-OC43, and HCoV-HKU1) (Perlman and Netland, 2009; Halaji et al., 2020). The genus β comprises of five subgenera where the entire bat derived coronaviruses fall. Further phylogenetic analysis showed that this 2019-nCoV hailed from the bat coronavirus reservoir, although during transmission to human, an intermediate host between bat and human is suggested (Lam et al., 2020; Lu et al., 2020). Being a β-coronavirus, the isolate was 88% identical to coronaviruses of bats that cause SARS (bat-SL-CoVZC45 and bat-SL-CoVZXC21), 82% identical to SARS-CoV and 50% identical to MERS-CoV (Cascella et al., 2020; Lu et al., 2020). The International Virus Classification Commission named it the SARS-CoV-2 (Li X. et al., 2020).

The SARS-CoV-2 has round to elliptical form (diameter range 60–140 nm) that often displays pleomorphy. Representing a typical coronavirus genome, the SARS-CoV-2 has a non-segmented, positive sense, single-stranded RNA (Cascella et al., 2020; Coronaviridae Study Group of the International Committee on Taxonomy of Viruses, 2020; Zhu et al., 2020). Though the phylogenetic analysis presented data supporting the similarity of SARS-CoV-2 with β-coronaviruses of bats, the new virus showed itself to be distinct from the previously known SARS-CoV and MERS-CoV. The conserved ORF1ab (replicase complex) was found to be <90% identical to other members of β-coronavirus (Zhu et al., 2020). Some of the genes of the SARS-CoV-2 shared <80% sequence identity to those of the SARS-CoV, but the sequence of amino acids in the seven conserved domains of replicase enzyme (ORF1ab) of SARS-CoV-2 showed 94.4% identity with that of SARS-CoV (Zhou P. et al., 2020). This finding ascertained that SARS-CoV-2 and SARS-CoV are belonging to the same species (Zhou P. et al., 2020).

## The Overwhelming Power to Infect: Analyzing the Capabilities of SARS-CoV-2

Repurposing its existence, the SARS-CoV-2 jumped from animal to human and then human to human through droplets from patient's sneeze or cough as well as through direct physical contact (Chang et al., 2020; Li Q. et al., 2020). In no time the virus spread rapidly to establish infection not only through symptomatic but also through asymptomatic carriers, SARS-CoV-2 exhibited high potential to be the cause of a pandemic (Chang et al., 2020; Munster et al., 2020). Studies in support of this outbreak being in a very high-risk category with regard to its spreading, have estimated the transmissibility of SARS-CoV-2 in terms of *R*_0_, the basic reproduction number. *R*_0_is the representative value of the average number of new infections caused by one infected patient in a population. For *R*_0_ < 1, an infected patient spreads virus to <1 person and this would lead to a fall in the strength of infection. Whereas, if *R*_0_ > 1, the contagion will be transmitted to more than 1 person and the outbreak will increase. The value of *R*_0_ is central to an infectious disease epidemiology (Liu et al., 2020). The estimated *R*_0_ of SARS-CoV-2 ranges from 2.24 to 3.58 while that of SARS-CoV and MERS-CoV ranges from 2 to 5 and 2.7 to 3.9, respectively (Zhao S. et al., 2020). SARS-CoV-2 was isolated from Vero E6 and Huh7 cells where cytopathogenic effects were clearly shown (Zhou P. et al., 2020).

This review deals with the very novelty of the SARS-CoV-2 based on the reports the features of viral architecture, its mode of action and host response.

## Unique Features in the SARS-CoV-2 Genome

**Figure 1:**
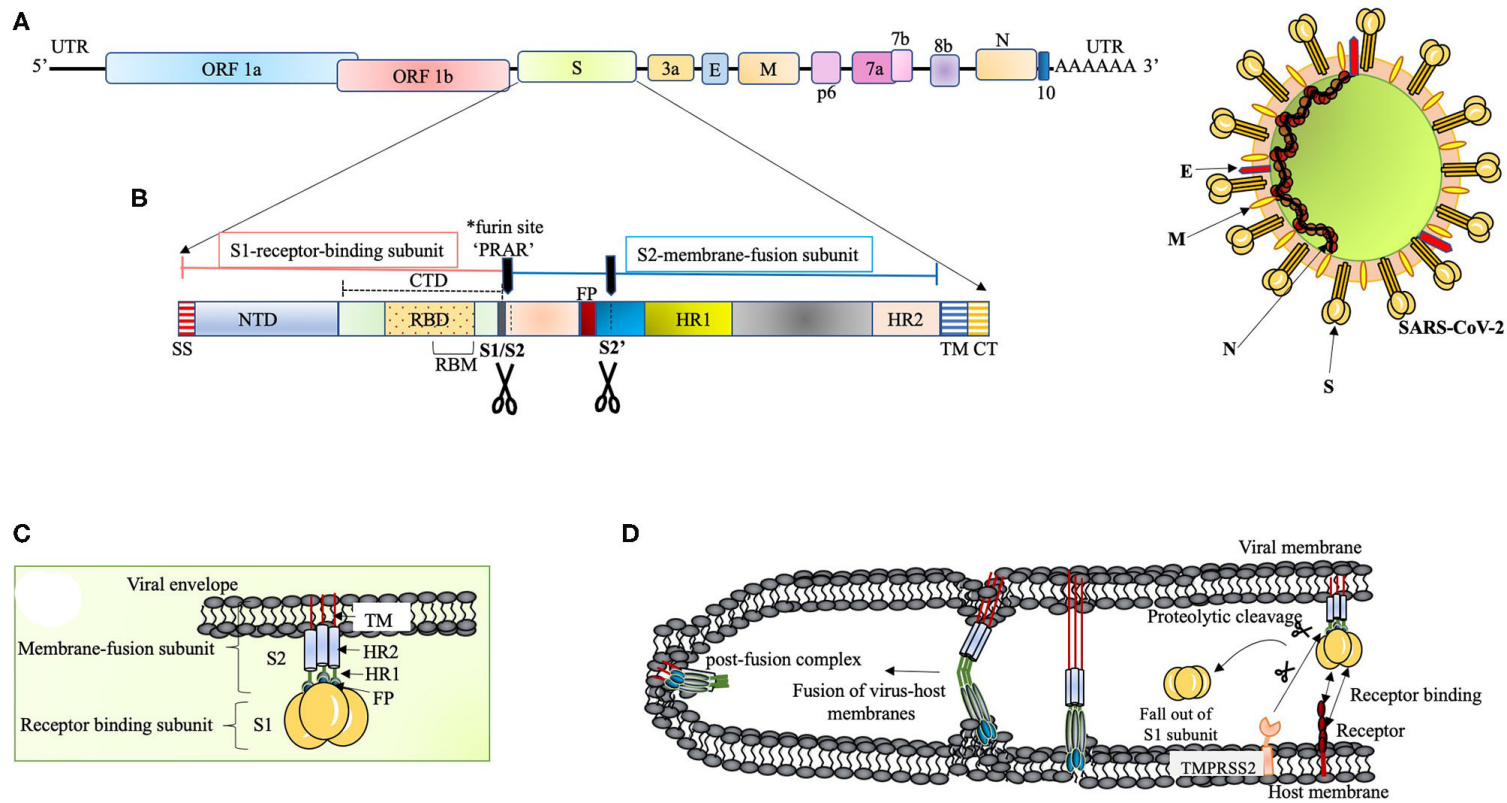
**(A)** The proposed genome organization of SARS-CoV-2. The RNA has 5' UTR followed by the predicted ORF 1ab encoding polyprotein ab (pp1ab) for 16non-structural proteins (NSP1 to NSP10 and NSP12 to NSP16). This segment is succeeded by: Spike glycoprotein (S), ORF3a, Envelope protein (E), Membrane protein (M), ORF6, ORF7a, ORF7b, ORF8, Nucleocapsid protein (N) and ORF10 (Kim et al., 2020). The ORF1ab polyprotein has site for (Bagdonaite and Wandall, 2018) cleavage by the virus coded 3CLpro and PLpro to form RNA-dependent RNA polymerase (RdRp) and helicase (Anand et al., 2003; Srinivasan et al., 2020). **(B)** The S polypeptide. The S1, receptor binding subunit, shows NTD and CTD differentiation where CTD harbors RBD with an internal RBM, which is conserved and recognizes ACE2. The S2, membrane fusion subunit, has fusion peptide (FP), S2' proteolytic site, two heptad-repeats, HR1 and HR2, and a transmembrane domain followed by cytoplasmic tail (CT) (Coutard et al., 2020). The S polypeptide is processed by extracellular proteases at S1/S2 site at the time of infection. The S protein has acquired a polybasic (*furin) site for cleavage at S1/S2 boundary, which is a unique feature of SARS-CoV-2 (Andersen et al., 2020; Walls et al., 2020). This feature widens the cell tropism of the virus (Kido et al., 2012). The polybasic site (PRRA) has a characteristic proline lead that has been predicted to invite addition of *O*-linked glycans (Andersen et al., 2020). **(C)** The S protein. The S protein (_~_1,200 aa long) is homotrimeric type I transmembrane protein. The S1/S2 cleavage site is predicted to acquire addition of *O*-linked glycans on the turn made by proline residue (PRRA) at the furin site. *O*-linked glycans have been studied to mask the immunodominant epitopes of viral antigens from immune recognition and augment virus-cell fusion through conformational changes (Bagdonaite and Wandall, 2018). **(D)** Proposed scheme of S protein and host receptor interaction. The S protein, upon binding with the host cell receptor, undergoes a step of priming wherein the extracellular proteases cleave off S1 subunit. This is followed by conformational changes in S2 subunit with the intercalation of FP within the host membrane. Finally, the HR1 and HR2 fold back to give post-fusion complex.

The genome organization of SARS-CoV-2 is discussed in Figure 1A. The 29,903 nucleotides RNA encodes structural proteins like spike protein (S), envelope protein (E), membrane protein (M), nucleocapsid protein(N), and accessory proteins, 3a, 6, 7a, 8, and 10 (Kim et al., 2020). The most alterable sequences in the coronavirus genome are harbored in the receptor-binding domain (RBD) of the S gene (Zhou P. et al., 2020). The *S* gene of SARS-CoV-2 has been studied to be longer than other reported SARS related coronaviruses (SARSr-CoV) (Zhou P. et al., 2020). This guided for the development of qPCR-based detection methods where the RBD, being the most variable sequence, was targeted (Zhou P. et al., 2020). Besides these data on RNA sequences, some significant differences in the amino acids of SARS-CoV and SARS-CoV-2 have been observed. The 8a protein was absent in SARS-CoV-2, the length of amino acids in 8b protein of SARS-CoV was 84 whereas that in SARS-CoV-2 was 121. The 3b protein of 154 amino acids in SARS-CoV was longer than that of SARS-CoV-2 displaying 22 amino acids (Wu et al., 2020). Worth noticing is the receptor-binding motif (RBM) in RBD that doesn't have any substitutions whereas other sequences of RBD harbor six mutations (Wu et al., 2020). Amino acid substitution study in other genes of SARS-CoV-2 found two important substitutions in NSP2 and NSP3 at the respective positions of 61 and 102 when compared with that of SARS-CoV (Wu et al., 2020).

These aspects merit further studies to understand the novel infectious capacity of SARS-CoV-2.

## The Viral Entry Into the Cell

The S protein of SARS-CoV-2 docks on the host cells by recognizing the receptor protein (a zinc-dependent carboxypeptidase), angiotensin-converting enzyme 2 (ACE2) as did the SARS-CoV (Li et al., 2003; Gheblawi et al., 2020; Zhang H. et al., 2020). ACE2 not only functions in the regulation of blood pressure but also against severe acute lung failure. The expression profile of ACE2 is wide. Apart from lungs, the other organ epithelial cells showing high expression levels of ACE2 are: bone marrow, brain, mouth, salivary glands, nasal lining, heart, thyroid, adipose tissue, gastrointestinal tract (duodenum, small intestine, colon, rectum), gallbladder, adrenal glands, kidneys, and male genital tissues (seminal vesicles and testis) (Li et al., 2020).

In the case of SARS-CoV, it was shown that the degree of lung injury was directly linked to ACE2 downregulation (Imai et al., 2005; Kuba et al., 2005; Sarzani et al., 2020). Studies have shown that the human ACE2 gets engaged with the S glycoprotein of SARS-CoV-2 with comparable degree of affinity as in the case of SARS-CoV. The sequence substitution study in RBD of SARS-CoV-2 discussed earlier, hinted that the interface for interaction with the host must be playing a crucial role in the viral tropism (Wu et al., 2020; Zost et al., 2020).

The S polypeptide and glycoprotein are discussed in Figures 1B,C, respectively. RBDs are the major determinants of host range and capacity to cross species barrier in coronaviruses (Li, 2015). It is therefore relevant to discuss NTD of the S1 subunit. It was proposed that coronaviruses stealthily inserted galactin gene (host lectin) in their S1 NTD, which resulted in their evolutionary divergence (Li, 2015). Conferred with the power of varying sugar-binding capacity, these viral lectins find their location in the cavities of the spike subunit whereby they can escape host antibodies during infection (Li, 2015). The S2 subunit (fusion-catalyzing domain, FDs) is the membrane-anchored component with necessary fusion machinery. After S1 undergoes proteolytic cleavage, the FDs get revealed through irreversible conformational transformations resulting in the intercalation of fusion peptide into the host membrane (Park et al., 2016) (Figure 1D). The presence of extensive N-linked glycans in the homotrimers of S protein not only help in gaining access to the host proteases for cleavage required for fusion but also attract neutralizing antibodies (Belouzard et al., 2009; Millet and Whittaker, 2014; Walls et al., 2017).

A concerted role of various host factors in the cell tropism of the virus is very important. The entry of SARS-CoV-2 into the cell is largely dependent on host protease activity, wherein the transmembrane protease, serine 2 (TMPRSS2), co-expressed with ACE-2 in the lung tissue, has been shown to facilitate the processing of the S protein (Matsuyama et al., 2005, 2010) (Figure 1D). The cleavage of the S protein by TMPRSS2 has been shown in augmenting SARS-CoV pathogenesis (Glowacka et al., 2011; Reinke et al., 2017; Hoffmann et al., 2020). Acting as a double-edged sword for the virus, this feature not only aids in the cleavage of S protein to facilitate virus entry but also interferes with neutralizing antibodies (Glowacka et al., 2011). This mode of direct entry through fusion with the host membrane was shown to be 100 times more effective than the endosome-mediated pathway of virus entry in SARS-CoV tropism (Matsuyama et al., 2005). Furthermore, the S protein has also been shown to associate with the C-type lectin expressed on the dendritic cells (DCs), where SARS-CoV infection is mediated *in trans* (Lau and Peiris, 2005).

## Harkening the Immunological Past to Decipher SARS-CoV-2

**Figure 2:**
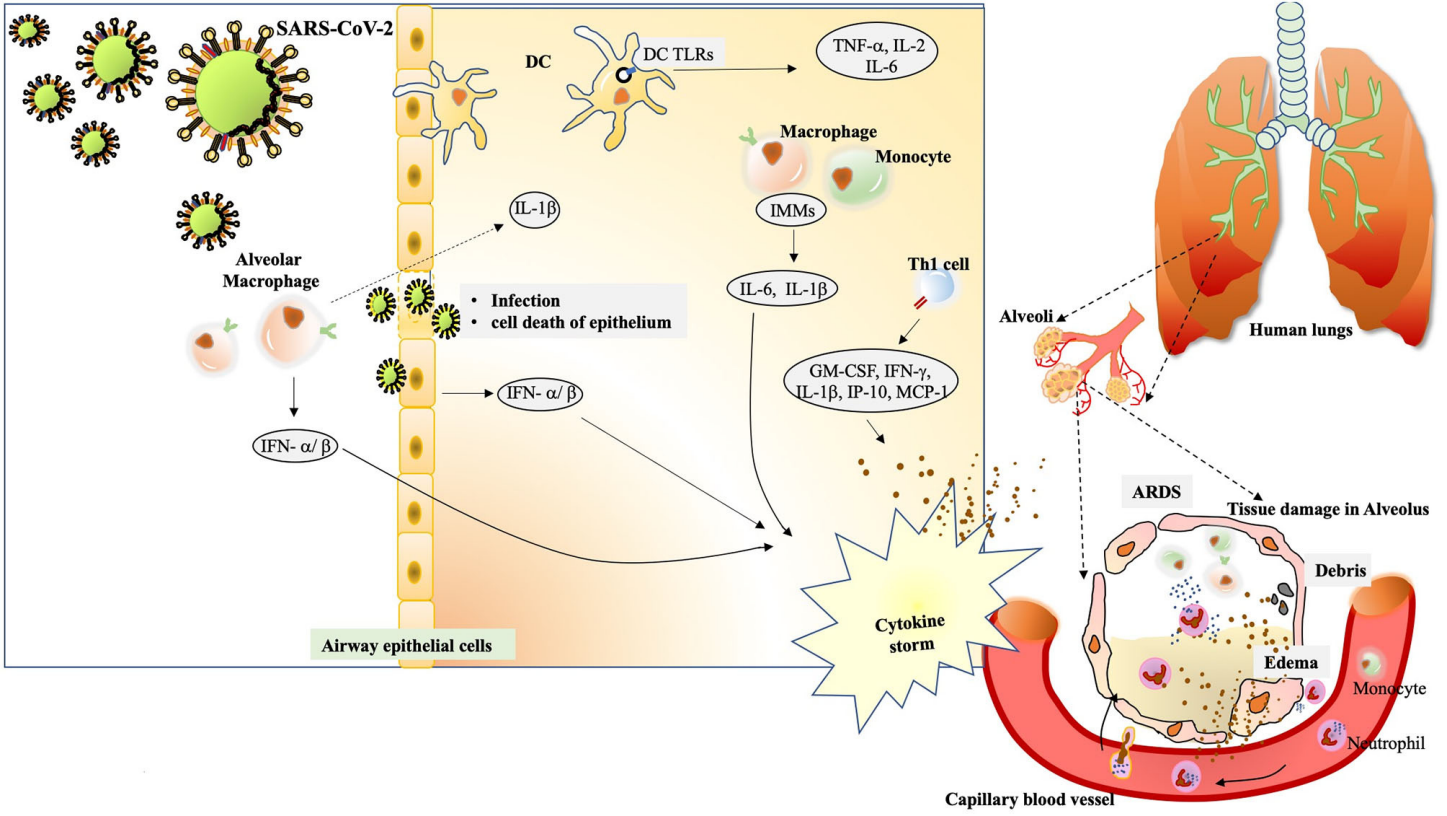
The immunological drama mastered by SARS-CoV-2. The alveolar macrophages act as first sentinels in the airways. The innate arm of immunity shows overzealous activation during severe cases of SARS-CoV-2.After confronting SARS-CoV-2, macrophage inflammasome-mediated release of IL-1βinduces a robust host response visible during immunopathology. At the same time, they induce vigorous expression of IFN-α/β that add on to the cytokine storm. Inside the alveolus, the DCs also stimulate expression of TNF-α, IL-2 and IL-6 through TLR signaling. The cytopathic effect of SARS-CoV-2 can be seen in the epithelial damage. Also, the infected cells, exerting their “by stander” effect, also induce IFN-α/β. Subsequent interaction of virus/antigens with IMMs further augments in the concentration of inflammatory cytokines. Th1 activation also charges a set of cytokines to add up to cytokine storm. The cytokine storm exerts damaging effect in alveolar tissue. The extravasation of circulating neutrophils and monocyte under the existing cytokine stimulation manifests in edema and deposition of debris in the alveolus. The condition of ARDS is conspicuously registered.

Nearly two decades back, the upsurge of SARS-CoV and then latterly on MERS-CoV, has provided a plethora of information on the pathophysiology and pathogenesis of the ever-evolving human coronaviruses. Many studies are emerging where labs are using the data acquired through previous research on SARS-CoV for understanding the behavior of SARS-CoV-2. The pathogenesis of SARS-CoV involves a complex network of events that not only manifests as severe injury to lungs but also a widened effect to other organs of the body. The immunological evaluations done on SARS-CoV clearly underline situations like high viral load, a storm of cytokines like CCL3/MIP-1α, CXCL10/IP-10, and CCL2/MCP-1, substantial infiltration of lung by macrophages and monocytes and the very fast diminishing levels of T cells (Chen et al., 2010). The condition of hypoxemic respiratory failure manifesting as acute respiratory distress syndrome (ARDS), is majorly contributing to mortality in SARS-CoV-2 infection. ARDS has been characterized as a systemic inflammation where bilateral involvement of lungs and other organs is evident. The modality of ARDS displays massive build-up of inflammatory cytokines like IL-1β, IL-6, TNF-α etc. in both broncho-alveolar lavage fluid (BALF) and plasma circulation (Figure 2). A non-resolving inflammation sets in where polarization of monocytes/macrophages is observed with the production of nitric oxide (NO), ROS and inflammatory cytokines that exert damaging effects on the lungs (Liu et al., 2019).

Here we present evidences of the multipronged immunological response during SARS-CoV and MERS-CoV pathogenesis and bring out its correlation with the work done on the present SARS-CoV-2 behavior.

## The Innate Defense

The working of innate immunity starts with the detection of the pathogen-associated molecular patterns (PAMP) (viral component or intermediates of replication) by the pattern recognition receptors (PRRs), wherein the Toll-Like Receptors (TLRs), the NOD-like receptors (NLRs) and the RIG-I-Like Receptors (RLRs) represent as significant PRRs against RNA viruses. The PRRs are ubiquitously distributed on the plasma membrane, endosomal membranes and in the cytosol (Arpaia and Barton, 2011; Rathinam and Fitzgerald, 2011). The sensing of viral PAMPs by these cellular arsenals (PRRs) charges a battery of programming that involves gene expression upregulation of inflammatory antivirals like cytokines, chemokines and interferons (IFNs) (Totura and Baric, 2012). These molecules being the “whistle blowers,” pave way for two important events: firstly, IFN-I induces a signaling network, where numerous IFN-stimulated genes (ISGs) are expressed and secondly the production of cytokines and chemokines stimulate neutrophils, macrophages, NK cells and DCs. In an infected lung, there is copious number of macrophages and DCs that function in controlling the infection via the production of cytokines and acting as antigen presenting cells (APCs). Being first respondents to the pathogenic invasion, their interaction with the virus becomes a significant parameter in the conclusion of infection. The maturation of DCs, stimulated by inflammatory cytokines like tumor necrosis factor alpha (TNF-α) and IL-1, is very important event in the immune response of a host during a viral infection. The priming of T cells by the DCs in the lymphatic organs becomes the ensuing event before a series of immune responses set in. Therefore, incapacitation of DCs becomes an excellent target for the virus whereby the very early step of immune response initiation is overcome during early infection cycle of the virus. The antigen presentation by APCs on their major histocompatibility complex (MHC class I) or precisely human leukocyte antigen (HLA) complex and then their recognition by virus-specific CD8+ cytotoxic T lymphocytes (CTLs) is a hallmark step in the initiation of host immune response.

The bystander effect of IFN-I on the unaffected cells neighboring infected cells, provides significant antiviral status, recruits innate immune cells and primes the adaptive immune response. The activation of the JAK/STAT pathway by IFN-I robustly regulates immune response network. Additionally, stimulation of DCs, NK cells and CTLs are also key to IFN signaling (Cristiani et al., 2020). All these molecules not only orchestrate the local fight against the virus but also the adaptive immune response. But situation specific cross-talks between DCs and NK cells could lead to the amplification in innate response strength (Andoniou et al., 2005).

## The Adaptive Defense

The innate arm of immunity is insufficient in not only clearing a posing infection but also registering memory for subsequent infections in the host (Flynn et al., 1998). The innate immune network passes on a set of instructions to the adaptive network, after deciphering the “non-self” from the “self” (Janeway, 1989). The convention of adaptive immune response begins with the uptake and sampling of the virus/its antigens by DCs, which further mature into APCs and migrate to draining lymph node system to educate T and B cells (Grayson, 2006). After developing in the bone marrow, immature DCs translocate to the peripheral tissues like lungs, where they gather antigens with the help of PRRs and get stimulated by several pro-inflammatory cytokines resulting in their maturation. The event marks down-regulation of PRRs, showing up of MHC, CCR7, CD80, and CD86 on their cell surfaces (Saeki et al., 1999).

The cell mediated immunity involves the action of CTLs, NK cells and macrophages. The recognition of viral antigens stimulate the expression pattern of various cytokines by T helper (Th) cells. The T-dependent activation of B cells for the production of antibodies against virus, is facilitated by the CD4+ T cells (Xu and Gao, 2004). The effects of CD8+ T cells are exerted in two ways: secretion of cytokines (IFN-γ, TNF-α, IL-2) as well as chemokines and cytolysis of the target cells (Frasca et al., 1998). The Ths, Th1 cells direct the activation of macrophages and Th2 cells stimulate eosinophils. On the other hand, the B cells, through their antibodies, regulate the complement pathway, phagocytosis and degranulation of mast cells.

The very critical action of CD8+ T cells in clearing the virus-infected cells, has been well-studied in mice (Doherty et al., 1997; Topham et al., 1997). The physiological role of CTLs in combating respiratory virus infection is also translated through the lysis of infected cells leading to tissue destruction (during eclipse phase of virus replication) (Zinkernagel and Althage, 1977). The condition of immunopathology is worsened in cases of cytopathic viruses where tissue damage is collaterally executed by CTL response to the virus (Reviewed in Zinkernagel, 1989).

### The Behavior of SARS-CoV

The SARS cases showed high serum concentrations of cytokines, neutrophils, and monocyte-macrophages in the lungs. This trend of extensive immunopathology arising out of severe inflammation has been studied to be a notable feature of other coronaviruses (Kindler and Thiel, 2016). In the context of HLA alleles, HLA-A24 being the most common HLA-A alleles, has been studied to be predominant in Eastern Asiatic region (Middleton et al., 2003). In the case of SARS-CoV, the HLA-A2-restricted CTL epitopes have been studied (Tsao et al., 2006; Kohyama et al., 2009; Ohno et al., 2009). The immunogenic region of N protein, which is an HLA-A^*^2402 restricted epitope, has also been studied to be a prominent candidate CTL antigen in the case of SARS-CoV (Liu et al., 2010). Previous studies have given a range of polymorphism in HLA in relation to susceptibility and protection from the SARS-CoV infection (Keicho et al., 2009; Wang et al., 2011).

Studies on SARS-CoV infected DCs have been of prime importance here. DCs are known to express a variety of receptors for pathogen recognition where TLRs of two kinds, the one present on the cell surfaces (TLR-1, TLR-2, TLR-4, TLR-5, TLR-6, TLR-10) and those expressed within intracellular compartments (TLR-3, TLR-7, TLR-8, TLR-9) are significant. The signaling cascade activation through TLRs results in DC induced production of costimulatory molecules like CD80 and CD86 as well as pro-inflammatory cytokines like TNF-α, IL-2, and IL-6 (Thoma-Uszynski et al., 2000; Kaisho and Akira, 2001). The signaling of TLR-7 in plasmacytoid DCs during SARS-CoV infection is antagonized by the Orf1a encoded PLPro (Papain-Like Protease) resulting in the diminishing of cytokine production and this acted against the establishment of antiviral response (Li et al., 2016). Other cellular targets antagonized by various SARS-CoV proteins are discussed in Table 1A. The activation of TLR-3 and TLR-4 through TRIF adapter protein imposes strong cell-intrinsic defense response against SARS-CoV infection (Totura et al., 2015). The use of TLR agonists have been well-projected as respiratory vaccine adjuvants (Zhao et al., 2012; Shirey et al., 2013; Perez-Giron et al., 2014). This prompted the usage of TLR-3 and TLR-4 agonists to have protection against SARS-CoV infection (Totura et al., 2015).

**Table 1A:** Cellular targets antagonized by various SARS-CoV protein.

**SARS-CoV**	**Proteins of the virus**	**Cellular targets**	**References**
1.	Orf3b	Direct inhibitor of IFN-β	Spiegel et al., 2005
2.	Orf3a	Suppression of IFN signaling (PERK pathway)	Minakshi et al., 2009
3.	Orf6	Inhibits IFN signaling by interfering STAT1	Frieman et al., 2007; Siu et al., 2009
4.	M and N	Block IFN-I production and NF-κB signaling	Kopecky-Bromberg et al., 2007; Siu et al., 2009
5.	NSP1	Inhibit IRF3-STAT1 and NF-κB pathways	Wathelet et al., 2007
6.	NSP3	Antagonizes IFN-β	Frieman and Baric, 2008

In the murine model of SARS-CoV, an increasing viral replication showed delayed response of IFN-I. The report showed milder or subclinical SARS in younger infected mice whereas aged mice developed a more severe version of the disease. This simulation was parallel to the development of SARS observed in humans where being “aged” guaranteed a severe form of the disease. IFN-I was not detected in BALF of mice until 24 h post infection, which potentiated the buildup of inflammatory monocyte-macrophages that release IL-6, IL-1β. This results in higher levels of cytokines, leakage in vasculature and abrogation in T cell response (Channappanavar et al., 2016). The serum build-up of inflammatory cytokines like IL-1β, IL-6, IL-12, Th1 cytokine, IFN-γ, and chemokine IP-10 were reported in SARS patients with overt pulmonary inflammation and lung damage. Additionally, there were substantial elevation of neutrophil chemokine IL-8 and monocyte chemoattractant protein-1 (MCP-1) (Wong et al., 2004). All of these observations unequivocally supported the overzealous innate response with massive infiltration of monocytes-macrophages and neutrophils in the SARS-CoV infection.

The intranasal administration of IFN prior to the peak reached during replication of virus displayed protective role in mice (Channappanavar et al., 2016). Paradoxically, IFN-I has been indicated to perpetuate fatal immunopathology by subverting the T cell response through inhibitory molecules like PD-1 and LAG-3. The study further attested that the lethality of SARS was not only contributed by TNF but also by the accumulation of IMMs (Channappanavar et al., 2016). Additionally, virus encoded proteins or the components of their replication have been well-documented as antagonists in the induction of IFN signaling. In this vein, SARS-CoV has been known to code proteins antagonizing RLR signaling (Shi et al., 2014).

The pro-inflammatory cytokines and chemokines produced in SARS-CoV infection were significant in numbers. Higher serum concentrations of TNF-α, IL-6, CCL3, CCL5, CCL2, CXCL10 were found in severe SARS patients (Channappanavar and Perlman, 2017). On the contrary, the levels of anti-inflammatory cytokines, IL-10 were low in severe cases of SARS-CoV (Chien et al., 2006).

The immunogenicity of the S protein showing the T cell epitopes were recognized for eliciting T cell response through the IFN-γ expression, in the convalescent SARS-CoV patients (Wang B. et al., 2004; Wang Y. D. et al., 2004). Alongside the S protein, the N protein of SARS-CoV also elicited persistent levels of anti-N antibodies and CD8+T cell response (Peng et al., 2006). The T cell mediated immune response against the N protein of SARS-CoV led to the generation of a strong population of IFN-γ producing T cells in animal studies (Zhao et al., 2005). The adverse cases of SARS-CoV infections showed lymphopenia with characteristic decline in both CD4+ as well as CD8+ T cells. However, there was a dramatic restoration of CD4+, CD8+ T cells, and B cells in patients who recuperated from SARS-CoV infection (Li et al., 2004).

The remarkable significance of virus-specific T cell mediated response was exemplified in the mouse model where a poorly stimulated immune system was shown to recover through the delivery of epitope-specific cultured CD8+ T cells (Zhao and Perlman, 2010).

The active role of humoral immunity is well-reported during SARS-CoV infection where the serum IgG, IgM, and IgA against viral antigens have been observed (Hsueh et al., 2004). The S protein has emerged as a strong candidate for immune protection through vaccination. The antigenic potential of the N protein is very high in SARS-CoV as seen in the convalescent sera of the patients (Zhong et al., 2005). The S1 domain of S protein has been tested to stimulate neutralizing activity against monoclonal antibodies (Sui et al., 2004). Nonetheless, the development of ARDS in SARS-CoV patients coincided with IgG seroconversion (Peiris et al., 2003). The presence of higher neutralizing antibodies (Nab) against S protein (IgG) and more binding antibodies against N protein, was recorded in recovered patients of SARS-CoV (Zhang et al., 2006; Seydoux et al., 2020). Albeit, the presence of Nabs in a study group showed a sudden rise in the serum of deceased patients as compared to patients who recovered. This behavior was in congruence with the deranged state of immune system with high level of inflammation leading to the systemic breakdown during SARS-CoV infection (Zhang et al., 2006). The average number of days for the peak activity of Nab in recovered patients was 20 days whereas that in deceased patients it was only 14.7 days. The comparative analysis during the same number of days, showed that Nab titers were much higher in deceased patients than in those who recovered (Zhang et al., 2006). The heterogenous macrophage population plays a significant role at various stages of SARS-CoV infection in anti-S IgG treated lungs. The non-resolving pro-inflammatory macrophages with lowering levels of TGF-β presented conditions of severe lung injury, whereas increasing TGF-β expression had connection with resolving macrophages as evidenced in mild infections (Liu et al., 2019).

The condition of antibody-dependent enhancement (ADE) of viral infection has been reported where the complexes of antibodies and viruses, formed via Fc receptor-mediated endocytosis, establish infection in monocytes and macrophages (Olsen et al., 1992). In support of this observation with respect to SARS-CoV infection, an abatement in the production of pro-inflammatory cytokines resulted after FcγRs blockage (Liu et al., 2019). The RBD of the S protein also displays neutralization epitopes, prime host immune response and can be a potential vaccine candidate (Li, 2015). Other proteins of SARS-CoV like M and E also elicited antibody responses (Jin et al., 2005).

### The Behavior of MERS-CoV

The role of HLA class II alleles have also been ascertained in cases of MERS-CoV infection (Hajeer et al., 2016). The S protein of MERS-CoV stimulates the expression of TLR negative regulators that impede viral clearance (Al-Qahtani et al., 2017; Mubarak et al., 2019). MERS-CoV infection studies also showed delayed but substantial response of all the three IFNs (type I, II, and III) (Menachery et al., 2014). Also, the infection witnessed an upsurge in pro-inflammatory cytokines and chemokines (IL-6, IL-1β, TNF-α, IFN-γ, and IL-8) (Lau et al., 2013). Here, higher levels of IL-8 were observed indicating the recruitment of neutrophils during MERS-CoV infection (Lau et al., 2013). The infection study also showed a detectable antiviral IFN-mediated response (Tynell et al., 2016). The MERS-CoV proteins antagonizing cellular targets are discussed in Table 1B. Data from a study on APCs, MDM (monocyte-derived macrophages) and MDDCs (monocyte-derived dendritic cells), which are residents of mucosal surfaces in the respiratory tract, showed that MERS-CoV readily established productive infection in human MDMs and immature MDDCs but failed to do so in the mature ones (Cong et al., 2018). Since immature MDDCs are efficient in uptake and processing of antigen whereas poor in stimulating T cells, this provides MERS-CoV with ample time for replication and thereafter dissemination for a successful productivity (Cong et al., 2018). Furthermore, clinical observations corroborated that the MERS-CoV showed systemic dissemination in patients as compared to SARS-CoV (Drosten et al., 2013). The chemoattractants, cytokines and chemokines were reported to be endurably produced during MERS-CoV infection that recruited immune cells in the lower respiratory tract leading to severe inflammation and hence tissue destruction. Lymphopenia and thrombocytopenia were also reported during MERS development (Zaki et al., 2012; Drosten et al., 2013). The role of CD8+ T cells and antibodies against the S protein of MERS-CoV, has been recognized as a protective candidate required for virus clearance in mice (Zhao et al., 2014). The RBD in the S1 domain of the S protein in MERS-CoV has also been shown to induce mucosal and systemic immune response (Li et al., 2019).

**Table 1B:** MERS-CoV proteins antagonizing cellular targets.

**MERS-CoV**	**Proteins of the virus**	**Cellular targets**	**References**
1.	Orf4a	Inhibition of IFN signaling	Yang et al., 2013
2.	Orf4b	Inhibition of IFN-I (NF-κB inhibition)	Matthews et al., 2014
3.	M, Orf5, Nsp3	Suppression of IFN-I	Yang et al., 2013, 2014
4.	Orf8b	Antagonizes IFN-β	Lee et al., 2019

Generally, dsRNA (formed after replication) acts as PAMP prototype in coronaviruses (Kindler et al., 2016). The entry of SARS-CoV has been shown to be restricted by IFN-induced transmembrane (IFITM) proteins (Huang et al., 2011). As studied in cell culture, IFN induction is diminished in both SARS-CoV and MERS-CoV infections (Kindler et al., 2016). SARS-CoV mouse model displayed pro-inflammatory monocyte-macrophage and cytokines that led to vascular leakage in lungs as a result of delayed induction of IFN despite the presence of higher levels of dsRNA (Kindler et al., 2016). This draws attention toward an important strategy for the virus in evading host antiviral response wherein both SARS-CoV and MERS-CoV stimulate the production of double-membrane vesicles (DMVs) acting as virus replication complexes that don't express any PAMPs (Kindler et al., 2016).

Mounting evidences show that the dysregulation of immune response and a surge in the production of chemokines and cytokines leads to a “cytokine storm” in the host, which has been delineated to be in congruence with the disease severity and poor prognosis, in both SARS-CoV as well as MERS-CoV (de Wit et al., 2016; Newton et al., 2016).

## A “NO Truce” Situation Between the Two Arms of Immunity

The condition of immunopathology arises when the invading pathogen awakens collateral damage due to the host immune response. The dynamism of host-virus relationship witnesses the attempts of a virus to eschew its visibility and the responses of the host through a myriad of challenges aiming at weakening the infection. The first line of defense, which combats the pathogen is innate immunity whereas reaction of adaptive response steps in after a few days. Infectious conditions resulting in an unleashed innate response with an absence of T cell supervision, leads to high damage in the host leading to death.

Studies on the comparative retrospection of SARS where patients who recovered against the ones who lost their lives, clearly support the fact that the innate arm of immunity cannot elicit a productive adaptive response, leading to succumbing of patients due to debilitating inflammation. SARS-CoV patients showed early expression of IFN-α, IFN-γ, CXCL10, and ISG-encoded proteins whereas only those who recovered exhibited adaptive immune response. Patients, who succumbed to the infection, had higher levels of CXCL10, CCL2, and ISG-encoded proteins with low levels of antibodies against the spike protein. It has been shown before with other viral diseases that the condition arising due to compromised gene regulation of HLA and antibodies leads to an aberrance in antigen presentation and production of antibodies. The same has also been documented during SARS-CoV infection (Cameron et al., 2007).

### The Behavior of SARS-CoV-2

The main phenotypic expression of SARS-CoV-2 is ARDS, a condition coordinated by cytokine release syndrome (CRS), that bears a high degree of similarity with SARS-CoV and MERS-CoV (de Wit et al., 2016). The hypercytokinemia, reported in SARS-CoV-2 has been saliently associated with upregulated expression of chemokines and their receptors. The chemokines ranking ahead are CXCL17 and CXCL8, which are known to be recruiting neutrophils into the lungs (Zhou Z. et al., 2020). The studies based on the pathophysiology of SARS-CoV and MERS-CoV have proposed targets for therapeutic interventions aiming at the initial stage of the SARS-CoV-2 infection, but the actual challenge faced is the extreme inflammation arising at latter stage of the disease where ARDS escalates mortality rate (de Wit et al., 2016). As endorsed in the cases of SARS-CoV and MERS-CoV infections, the infliction of conditions like pneumonia and “cytokine storm” has been again known to be the underlying cause of cellular destruction in the host of SARS-CoV-2 (Chen G. et al., 2020; Chen N. et al., 2020). The testimony supporting the devastations caused during SARS-CoV-2 infection because of the immunopathogenesis can be well-witnessed in a number of reports (Channappanavar and Perlman, 2017; Chen G. et al., 2020; Chen N. et al., 2020; Chua et al., 2020; Giamarellos-Bourboulis et al., 2020; Guan et al., 2020; Huang et al., 2020). The clinical condition of SARS-CoV-2 infection is driven by dysfunctional immune system where profound lymphopenia, sepsis due to macrophage-activation and stumpy expression of HLA-DR on CD14-monocytes accompanied by excessive accumulation of IL-6 and IL1RA are observed (Giamarellos-Bourboulis et al., 2020). The presence of monocyte-associated chemokines (CCL2 and CCL8), appearance of circulating neutrophils and delayed expression of IFN-1, all are suggestive of the response to SARS-CoV-2 infection in the new human host (Bianco Mello et al., 2020). Going against the characteristic feature of SARS-CoV, the SARS-CoV-2 induces a robust IFN response, which is attributed to be the contributing factor for not only a higher percentage of milder or asymptomatic cases but also lower mortality rate (Zhou Z. et al., 2020). In severe cases, IFN-I expression could be at times be non-redundant or morbific, especially in epithelium tissues (Channappanavar et al., 2016; Ziegler et al., 2020). In agreement with this observation, cytokine treatment resulted in IFN-α driven expression of ACE-2, which could facilitate the enhancement of SARS-CoV-2 infection (Ziegler et al., 2020). The lung injury imposed during SARS-CoV-2 infection with reports of increased neutrophils and lowering lymphocyte counts clearly indicate about a possible interplay between virus PAMPs and host PRRs (Prompetchara et al., 2020; Tay et al., 2020). The innate arm of immunity mediated by RLRs gets activated by RNA 5'-triphosphate, hence addition of modified residues on 5'-triphosphate group abrogates RIG-1 activation. The NSP16/NSP10 heterodimer of SARS-CoV-2 has been reported to perform 2'-O methylation of the first nucleotide of its mRNA (Viswanathan et al., 2020). This feature would not only be helping in efficient and higher rate of viral mRNA translation but also evading recognition by PRRs. Similarly, the stress granule proteins (SGs) are known to provide scaffold for RLRs and virus mRNAs that stimulate the IFN pathway (Nakagawa et al., 2018). The N protein of SARS-CoV-2 has been shown to interact with SGs that disrupts the latter thereby affecting IFN signaling (Gordon et al., 2020).

There is a notable reduction in the circulating lymphocytes and CD4+ as well as CD8+ T cells, specifically in severe cases of SARS-CoV-2 infection (Chen G. et al., 2020; Peng et al., 2020). However, the hyperactivation status of CD4+ and CD8+ T cells showed high HLA-DR on the latter. The levels of differentially expressed IFN-γ and TNF-α in the CD4+T cells dropped down in severe cases as compared to milder infections of SARS-CoV-2. At the same time, perforins and granzyme B in CD8+T cells rose to higher levels in severe cases than those in milder cases. These observations have been implicated in the damage caused by SARS-CoV-2 resulting in the lowering of antiviral immunity in the host (Zheng H. Y. et al., 2020).

The Th1 cells have been shown to get activated resulting in the generation of granulocyte-macrophage colony-stimulating factor (GM-CSF), IFN-γ, IL-1β, IP-10, and MCP-1 (Huang et al., 2020). On the contrary to SARS-CoV, SARS-CoV-2 infection showed elevated levels of IL-4 and IL-10 cytokines from Th2 cells (Huang et al., 2020). The presence of macrophage inflammatory protein 1α (MIP1A) and TNF-α are also significant in SARS-CoV-2 infection (Zhang Y. et al., 2020). The cytotoxic granules harbored in CD8+ T cells were high (Xu et al., 2020). Severe cases have been shown to have higher cytotoxic follicular helper (T_FH_) cells as well as CD4+-CTLs in comparison to milder cases (Meckiff et al., 2020). The sera of convalescent COVID-19 patients show strong T cell response (Neidleman et al., 2020). Lymphopenia is seen commonly and has been held as a critical factor in disease severity (Weiskopf et al., 2020; Yu et al., 2020). Patients with severe pulmonary inflammation had higher levels of virus induced expression of NKG2A/CD94 (NK group 2-member A, an NK inhibitory receptor) leading to the exhaustion of NK cells and CTLs (Zheng M. et al., 2020).

The patients of SARS-CoV-2 showed escalated viral titres in the first week that showed gradual decline over the second week. The overt symptoms accompanied with ascension of IgG and IgM antibodies against N and S (RBD) protein of SARS-CoV-2, was evident around the 10th day of illness (To et al., 2020; Wang et al., 2020). The high viral titers have been implicated in several practical outcomes during the early phase: infectivity potential of the patient becomes high (high transmissibility) and the virus gains an advantage of evading antiviral defenses within the host (Chen and Li, 2020). As observed in SARS-CoV cases, despite showing neutralizing antibodies in the serum, a small fraction of patients faced persistent inflammation eventually succumbing to the infection. This phenomenon was explained by ADE during virus infection (Fu et al., 2020). The same observation can be extrapolated in SARS-CoV-2 severe cases where patients suffer damaging inflammation even though neutralizing antibodies are secreted (Okba et al., 2020). Potent antibodies against S protein that bind specifically to RBD interface, was also shown to impede interaction of SARS-CoV-2 with ACE2 (Wang et al., 2020).

Table 2 represents various cellular targets of SARS-CoV, MERS-CoV, and SARS-CoV-2.

**Table 2:** Host cellular targets of various SARS-CoV, MERS-CoV and SARS-CoV-2 proteins.

**Host target proteins**	**SARS-CoV proteins**	**MERS-CoV proteins**	**SARS-CoV-2 proteins**	**Stage of viral life cycle and function**	**References**
ACE2	S		S	Attachment and entry Host cell receptor	Weiss and Leibowitz, 2011; Hoffmann et al., 2020; Lan et al., 2020; Valencia et al., 2020
DPP4		S		Attachment and entry Host cell receptor	Li, 2015; Seys et al., 2018; Valencia et al., 2020
IFITM	Known	Known	Not known	Entry restricted	Wrensch et al., 2014; Liao et al., 2019
Cathepsin L	S	S	S	Cleavage and activation	Kleine-Weber et al., 2018; Ou et al., 2020
Furin		S	S	Cleavage and activation	Coutard et al., 2020; Xia et al., 2020
TMPRSS2	S	S	S	S protein priming	Kleine-Weber et al., 2018; Iwata-Yoshikawa et al., 2019; Hoffmann et al., 2020; Meng et al., 2020
GSK3	N			Phosphorylation Facilitation of viral replication	Wu et al., 2009
IFN pathway			NSP13, NSP14,NSP15 ORF6 and ORF9b	Antagonize interferon pathway	Lei et al., 2020; Sa Ribero et al., 2020
NF-κB	N		NSP13, ORF9c	Inflammation	Liao et al., 2005; Dosch et al., 2009
E3 ubiquitin ligase: TRIM59 and MIB1			ORF3a and NSP9, respectively	Interference with antiviral innate immunity	Kondo et al., 2012; Gil et al., 2020
E3 ubiquitin ligase: RCHY1	NSP3	PLPro		Abolishing of p53 mediated antiviral activity	Ma-Lauer et al., 2016; Gordon et al., 2020
CAMK2D	NSP3			Interference in IFN pathway	Ma-Lauer et al., 2016
NUP98-RAE1			ORF6	Antagonize interferon pathway	Addetia et al., 2020; Gordon et al., 2020
Stress granule proteins: G3BP1 and G3BP2, LARP1, CK2, UPF1, MOV10			N	Abrogation of IFN signaling	Cascarina and Ross, 2020; Gordon et al., 2020
Stress granule proteins		p4a interacts with dsRNA		Inhibition of stress granule formation	Rabouw et al., 2016; Nakagawa et al., 2018
*N*-linked glycosylation enzymes	S and M	S	S	Facilitation of lectin-mediated virion attachment by S	Zhou et al., 2010; Watanabe et al., 2020; Zhao P. et al., 2020
Caveolin	ORF3a			Might regulate virus uptake and trafficking of viral structural proteins	Padhan et al., 2007
TRAF3 and ASC	ORF3a			Activation of NLRP3 inflammasome	Siu et al., 2019
RUNX1b	ORF3b			Immunomodulation	Varshney et al., 2012
KPNA2	ORF6			Modulation of host protein nuclear transport and IFN-1 signaling	Frieman et al., 2007
KPNA4 (importin-α3)		p4b		Evasion of innate response	Canton et al., 2018
Bcl-xL	ORF7a, E			Induction of apoptosis Lymphopenia	Tan et al., 2007
LFA-1	ORF7a			Attachment factor on leukocytes	Hänel and Willbold, 2007
Calcineurin/NFAT pathway	NSP1			Induction of IL2 Immunopathogenesis	Pfefferle et al., 2011
PHB1 and PHB2	NSP2			Might be altering cell cycle progression, cellular differentiation, mitochondrial biogenesis	Cornillez-Ty et al., 2009
dsRNA		p 4a		Sequestration of dsRNA, suppression of PKR-dependent translation, suppression of RIG-I and MDA5	Rabouw et al., 2016
Polyprotein-cleaving protease activity	PLPro (NSP3)	PLPro (NSP3)	PLPro (NSP3)	Deubiquitination Antagonize innate immunity	Grum-Tokars et al., 2008; Clementz et al., 2010; Fung and Liu, 2019
PALS_1_	E			Breaching of alveolar wall	Teoh et al., 2010
Na^+^/K^+^ ATPase α-1 subunit and Stomatin	E			Reduction in activity of epithelial Na channel	Nieto-Torres et al., 2014; Schoeman and Fielding, 2019
hnRNPA1	N			Might regulate viral RNA synthesis	Shi and Lai, 2005
Cyclophilin A	N			Interferon pathway Might be crucial for virus infection	Yurchenko et al., 2010; Tanaka et al., 2013

*ACE2, Angiotensin-converting enzyme; DPP4, Dipeptidyl-peptidase; IFITM, Interferon-induced transmembrane protein; GSK3, Glycogen synthase kinase; TRIM59, Tripartite motif-containing protein; MIB1, Mindbomb E3 ubiquitin protein ligase; NUP98, Nuclear pore complex protein; RAE1, Ribonucleic acid export; G3BP, Ras-GTPase-activating SH3 domain binding protein; mTOR-regulated translational repressor LARP1, La ribonucleoprotein; CK2, casein kinase; mRNA decay factors UPF1, Up-frameshift) and MOV10, Moloneyleukemia virus 10 like; NFAT, Nuclear factor of activated T-cells; IL2, Interleukin; PKR, Protein kinase R; PLPro, papain-like protease; Bcl-xL, B-cell lymphoma-extra large; PALS_1_, Proteins associated with Lin seven; hnRNPA1, Heterogenous nuclear ribonucleoprotein; RUNX1b, Runt-related transcription factor; KPNA2, karyopherin alpha 2; LFA-1, lymphocyte function-associated antigen 1; PHB,),TRAF3, TNF receptor-associated factor3; ASC, apoptosis-associated speck-like protein containing a caspase recruitment domain; NLRP3, NOD-,LRR-and pyrin domain-containing protein),RCHY1, ring-finger and CHY zinc finger domain-containing 1; CAMK2D, calcium/calmodulin-dependent protein kinase II delta*.

## SARS-CoV-2: Unleashing the Trend of Age Bias

When the SARS epidemic emerged, the overall rate of mortality escalated on account of the lowering survival rate (50%) in patients above the age of 65 years. On the contrary, a marked disparity was seen in young patients (below 24 years) where survival rate was 100% (Peiris et al., 2004). Similar case was observed in MERS, where 45.2% mortality rate was observed in patients above the age of 60 years otherwise it was 20% for those under 60 years of age (Ahmed, 2017). In both the outbreaks of SARS-CoV and MERS-CoV, the existence of comorbidity in these patients elevated the risk of fatality with age (Chan et al., 2003; Yang et al., 2017). In the current pandemic of SARS-CoV-2, the susceptibility of patients with comorbidities lead to their poorer clinical outcomes (Guan et al., 2020; Mao et al., 2020).

ACE2 being one of the host membrane receptors of SARS-CoV-2, is known to be a negative regulator of angiotensin 2 (AngII), which raises blood pressure (Deshotels et al., 2014). The entry of SARS-CoV mediated by ACE2 has been shown to endocytose the receptor along with the virus after fusion, which results in diminished numbers of ACE2 receptors on the membrane leading to subsequent rise in serum levels of AngII (Kuba et al., 2005). The condition of ARDS has been well-known to cause acute lung damage where downregulation of ACE2 is in congruence with this model (Zhang and Baker, 2017). AngII has also been implicated in the immune cell differentiation and production of pro-inflammatory cytokines (Satou et al., 2018). The establishment of inflammation mediated via activation of NF-κB and IL-6 has been seen in the case of aged SARS-CoV patients (Brasier, 2010; Dediego et al., 2014). Since IL-6 has effect on cellular senescence, the rising levels of IL-6 in higher age cases of SARS-CoV-2 might correspond to their mortality (Hirano and Murakami, 2020). The expression of Th1 chemokines (CXCL9/10/11) and granzyme B have been shown to be at reduced levels in elderly patients of SARS-CoV-2 (Lieberman et al., 2020). The vaccine based preventive measures in aged population is an arduous task because of the poorer response in the older individuals (Katz et al., 2004; Kovacs et al., 2009). An investigation on the efficacy of SARS-CoV vaccine on aged mice also showed a waning effect (Deming et al., 2006).

As the pandemic has proceeded to establish the infection of SARS-CoV-2 in more and more individuals across the globe, there have been surprising reports wherein previously healthy young adults are showing severe COVID-19 infection. In a small cohort study, it was shown that lymphopenia could be a potential predictor of severe prognosis in younger adults with SARS-CoV-2 infection (Zhou C. et al., 2020). The reason for this observation still eludes us. Nevertheless, a few presumptions like overwhelming of the immune system by a sudden invasion of virus or individual specific immune response could explain this ambiguity where previously healthy immune systems are presenting ARDS. Another cross-sectional study reported younger patients with COVID-19 (median age 60 years) requiring intensive care unit (ICU) admission (Blake et al., 2020). It is surprising to note that the upper respiratory tract of both symptomatic as well as asymptomatic patients harbored similar viral loads (Zou et al., 2020).

The peripheral blood lymphocytes in children with SARS-CoV-2 infection are mostly within the range of normalcy, which is indicative of a controlled immune response (Cao et al., 2020).

However, one report from North American pediatric ICU has described COVID-19 infection severity in infants and children (~80% had comorbidity) (Shekerdemian et al., 2020). Though the burden of the disease in children was lower in comparison to that of adults, this can't let us ascertain that SARS-CoV-2 spares this section of the population.

The SARS-CoV-2 seems to be breaking the rule of age bias that was seen in previous infections of SARS-CoV and MERS-CoV. The trend is of great concern because the generally younger population who tend to remain asymptomatic sources of transmission, risking the elder groups, could also fall prey to the severity of COVID-19 (Liao et al., 2020).

## Future Prospects and Conclusion

SARS-CoV-2 has gripped the globe with its relentless capacity for transmission. After the outbreaks of SARS-CoV and MERS-CoV in the past two decades, the emergence of a third coronavirus, SARS-CoV-2 has not only over-burdened our global health system but also shaken up our pride of conquering all aspects of life. In the case of SARS-CoV, the outbreak was impeded because the symptoms became evident before a patient became infectious. This made the containment of individuals easier. However, increasing lines of evidences have shown that most of the SARS-CoV-2 patients remain asymptomatic carriers. The data repertoire of studies conducted on SARS-CoV is germane to the explorations in the field of SARS-CoV-2 pathogenesis and treatment. The SARS-CoV antigens were reported to be present in organs like liver, pancreas, kidneys and cerebrum as well as bronchi, lungs and intestine (Matsuyama et al., 2005). The severe tissue damage coalescing with SARS, to some extent, has been suggested to be due to the proteases secreted in the target organs of SARS-CoV infected patients (Matsuyama et al., 2005). Apart from the aforementioned proteases, elastase secreted by neutrophils have been proposed to aid in the fusion of SARS-CoV envelope with the host membrane via cell-surface-mediated entrance. This has been subsequently known to enhance virus infection as compared to the canonical endosomal pathway of their entry (Matsuyama et al., 2005).

SARS-CoV and MERS-CoV emerged with a contagious advantage where they diminished the response of innate immunity in the host. One therapeutic approach here would be the use of agonists of the innate pathway that could reinstate antiviral state in the host. The proposal to use agonists as well as antagonists of TLRs has been a good choice that displays a broad-spectrum potential in therapeutics against some respiratory infections (Zhao et al., 2012; Shirey et al., 2013; Perez-Giron et al., 2014). In this line of evidence, data shows that the use of a hybrid IFN (IFN-α B/D) and controlled activation of TLR-3 by rintatolimod (Ampligen, poly I:poly C124), confers protection in cells/animals against immunopathology associated with cytokine storm (Barnard et al., 2006; Zhao et al., 2012; Perez-Giron et al., 2014). TLR-3 agonist, poly (I:C), has been shown to be protective against both SARS-CoV as well as MERS-CoV infection models (Zhao and Perlman, 2010; Zhao et al., 2014). Therefore, studies deciphering the role of PRRs in establishing antiviral state or identifying the points where virus antagonizes/escapes various PRR cascades, would deepen our knowledge on virus pathogenesis.

The gene polymorphism in the NFKBIA promoter (of gene NFKBIA, which codes for IκBα) has been shown to influence the innate arm of host immunity in various infections (Ali et al., 2013). A network-based analytical study on comparison between SARS-CoV and MERS-CoV infections strongly advocated a logarithmic scale upregulation in the gene expression of NFKBIA, which has been proposed to be a key regulator in the level of host immune response during virus infection (Moni and Lio, 2014). Therefore, studies on the genetic polymorphisms of NFKBIA in the current situation of SARS-CoV-2 infection could also help in understanding their impact on innate immunity.

Interestingly, SARS-CoV was shown to possess a single ORF8 during its earlier stage of spread, while the middle and late phases presented isolates with two fall outs of ORF8, i.e., 8a and 8b due to a 29-nucleotide deletion (Oostra et al., 2007). SARS-CoV ORF8b is known to activate NLRP3 inflammasome in macrophages accompanying the release of IL-1β (Shi et al., 2019). Hence, the presence of only ORF8b in SARS-CoV-2 can be focused in understanding the cause behind the ardent inflammation during infection.

The occurrence of various strains of SARS-CoV as well as MERS-CoV on account of the genetic diversity has raised concerns over the dimension and efficiency of potential vaccines (Consortium, 2004; Yang et al., 2005). The RNA viruses display higher rates of mutation, that selectively increases virus virulence, helps in escaping host defense and alters their tissue tropism (Consortium, 2004). A positive selection was seen in the case of SARS-CoV ORF 1a and S gene sequences, which showed most of the substitutions. Whereas, ORF1b was found to be the most conserved sequence of SARS-CoV (Consortium, 2004). In the same course of studies on the evolving nature of SARS-CoV, anti-S antibodies were also shown to bolster the entry of virus rather than neutralization (Yang et al., 2005). These opinions should be considered in further studies on the heterogenous infectious potential of SARS-CoV-2, which needs closer examination to understand the sequelae of infection.

A disturbed and exuberant innate immune response can cause devastating immunopathological condition in the host whereby our own immune cells, which are educated to fight against virus infections, cause immense destruction in the body tissues and organs. A perpetual evolution in the adaptive capabilities of the virus is highly dependent on the ever-changing environment and host behavior. Severity of a viral disease is in constant conflict with the need for the virus to be able to disseminate within the host. It evolves and adapts in its host. Hitherto, the virus provides its host with opportunities for clearance, but with the condition of keeping their transmission uninterrupted. As seen in the clinical course of SARS-CoV infection, three distinct disease phases were characterized. In the first phase, virus replication occurs robustly manifesting fever and cough, which subsides in a few days. The second phase, despite showing a progressive lowering in viral titers toward the end, exhibits high fever and hypoxemia resulting into pneumonia-like condition. The third phase sees the patients developing ARDS, often leading to death (~20%) (Channappanavar and Perlman, 2017). As evident from the gradual decline in virus titer toward the very end of these clinical phases, the role of a completely dysregulated immune system resulting in a hyper state of inflammation in the host system.

Given the advancements in the field of research analytics and an ever-escalating data repertoire, the long-term sequelae of these coronavirus infections are still unforeseen. The increasing human globalization is now paying for the trajectory of progressiveness. Lastly, we will have to stop and think, whether we again want to become a target for the next pandemic virus.

## Author Contributions

RM and JK conceived the idea. All authors contributed equally in generating the draft and final versions of the manuscript.

## Conflict of Interest

The authors declare that the research was conducted in the absence of any commercial or financial relationships that could be construed as a potential conflict of interest.
